# Scalable, high performance, enzymatic cathodes based on nanoimprint lithography

**DOI:** 10.3762/bjnano.6.142

**Published:** 2015-06-22

**Authors:** Dmitry Pankratov, Richard Sundberg, Javier Sotres, Dmitry B Suyatin, Ivan Maximov, Sergey Shleev, Lars Montelius

**Affiliations:** 1 Biomedical Sciences, Health and Society, Malmö University, 20560 Malmö, Sweden; 2 A.N. Bach Institute of Biochemistry, 119071 Moscow, Russia; 3 Division of Solid State Physics and The Nanometer Structure Consortium (nmC@LU), Lund University, 22100 Lund, Sweden; 4 Neuronano Research Center, Medical Faculty, Lund University, 221 00 Lund, Sweden; 5 Kurchatov NBICS Centre, National Research Centre "Kurchatov Institute", 123182 Moscow, Russia

**Keywords:** bilirubin oxidase, bio-electrocatalysis, direct electron transfer, nanoimprint lithography, oxygen reduction reaction

## Abstract

Here we detail high performance, enzymatic electrodes for oxygen bio-electroreduction, which can be easily and reproducibly fabricated with industry-scale throughput. Planar and nanostructured electrodes were built on biocompatible, flexible polymer sheets, while nanoimprint lithography was used for electrode nanostructuring. To the best of our knowledge, this is one of the first reports concerning the usage of nanoimprint lithography for amperometric bioelectronic devices. The enzyme (*Myrothecium verrucaria* bilirubin oxidase) was immobilised on planar (control) and artificially nanostructured, gold electrodes by direct physical adsorption. The detailed electrochemical investigation of bioelectrodes was performed and the following parameters were obtained: open circuit voltage of approximately 0.75 V, and maximum bio-electrocatalytic current densities of 18 µA/cm^2^ and 58 µA/cm^2^ in air-saturated buffers versus 48 µA/cm^2^ and 186 µA/cm^2^ in oxygen-saturated buffers for planar and nanostructured electrodes, respectively. The half-deactivation times of planar and nanostructured biocathodes were measured to be 2 h and 14 h, respectively. The comparison of standard heterogeneous and bio-electrocatalytic rate constants showed that the improved bio-electrocatalytic performance of the nanostructured biocathodes compared to planar biodevices is due to the increased surface area of the nanostructured electrodes, whereas their improved operational stability is attributed to stabilisation of the enzyme inside nanocavities.

## Introduction

Reduction of oxygen (O_2_) is the key reaction in many natural and artificial systems, and indeed, this reaction is one of the most interesting research issues in both academia and industry today due to its importance in fuel cell technology [1]. The development of new, low cost, high activity catalysts for O_2_ reduction is under intensive investigation, with a special focus on renewable biological catalysts, such as living cells, organelles, and different redox enzymes, especially multicopper oxidases (MCOs) [2–3]. In nature, MCOs catalyse the oxidation of many organic and inorganic compounds (electron donors) using O_2_ as the only electron acceptor [4]. The O_2_ bio-electroreduction mechanism involves electron transfer from the electrode to the T1 copper (Cu) site with the concomitant reduction of O_2_ directly to H_2_O in a trinuclear Cu cluster (T2/T3 Cu cluster) positioned 12–13 Å away [5–7], without releasing reactive O_2_ species, such as hydrogen peroxide or superoxide radicals [8–9]. Three-dimensional electrodes for enzyme-based cathodes are usually used to design high performance biodevices based on MCOs and different nanomaterials [10–14]. Several techniques for reproducible fabrication of well-ordered porous electrodes have been already reported. The techniques are based on electrodeposition of metals using colloidal templates [15] or gas bubbling [16]. Nevertheless, the simple and irreproducible immobilisation of separately synthesised nanomaterials on electrode surfaces is typically achieved. This approach cannot be directly used on an industrial scale and renders one of the main general problems of bioelectronics, which hinders real, practical application of biodevices including biocathodes.

Here we show fabrication of nanostructured electrodes using nanoimprint lithography (NIL), which ensures the well-controlled nanostructured geometry of electrodes on an industrial scale. To the best of our knowledge, this is one of the first reports concerning the application of NIL for amperometric bioelectronics. NIL is a parallel patterning technique capable of rendering features as small as 2–3 nm (or even smaller) in a fast, reproducible, scalable and economical way [17]. Nanoimprinting is based on the pattern transfer by a replication technique where nanometer-sized features of a hard stamp (mould) are copied into a polymer layer by either a thermal or an UV-light imprint process. The very high resolution of nanoimprinting can be combined with printing on large areas (6 inches and larger) and industry-scale throughput. In our studies, we used a well-known and commercially available MCO, bilirubin oxidase (BOx), which is one of the main biocatalysts exploited today to design third-generation (i.e., direct electron-transfer-based), O_2_ reducing biodevices (e.g., O_2_-sensitive biosensors [18] and biocathodes of enzymatic fuel cells [19]). Contrary to many other MCOs, BOx is bio-electrocatalytically active when physically adsorbed on bare gold (Au), and the adsorption process is more or less irreversible [20]. Moreover, BOx-based biocathodes are highly active and stable across broad pH (5–8) and temperature (0–40 °C) ranges [5,21] in the presence of usual inhibitors of MCOs (e.g., halide ions [22]). Thus, we show one possible way to easily and reproducibly design (with industry-scale throughput) highly active and stable nanostructured biocathodes, which in principle can operate in solutions of different composition, temperature, and pH.

## Results and Discussion

**Figure 1:**
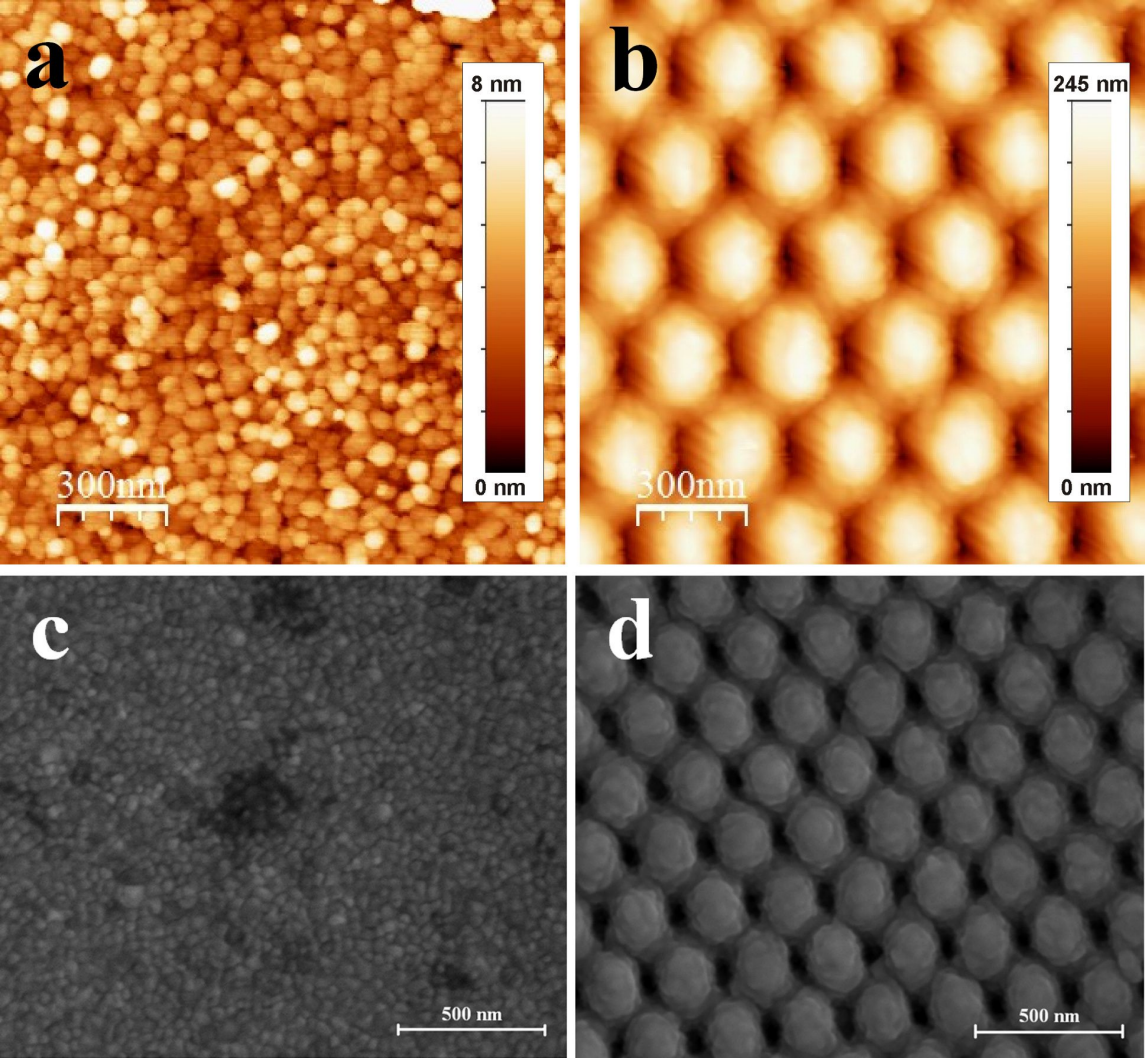
AFM (a, b) and SEM (c, d) images of a Au electrode (a, c) and a NIL/Au electrode (b, d).

Firstly, planar (control) and nanostructured (using NIL) Au electrodes (Au and NIL/Au electrodes, respectively) were fabricated. As control studies, electrochemically cleaned Au electrodes were imaged using AFM and SEM (Figure 1). According to AFM, the Au electrodes had a granular surface, in good agreement with previous reports [20,23]. Specifically, the grains exhibited lateral dimensions in the range of 20–80 nm, and vertical dimensions in the 1–8 nm range, resulting in a roughness factor (*f*) of the Au surface equal to 1.06 ± 0.02 (Figure 1a, left). Indeed, for Au electrodes *A*_real_ was only 1.12-fold higher compared to *A*_geom_. It is clearly seen that the NIL modification resulted in the formation of a regular, well-ordered, 2D hexagonal lattice of nanocavities, increasing the *f* value to 1.65 ± 0.03 (Figure 1a, right). The centres of the nanocavities were separated by an average distance of approximately 300 nm, whereas their depth was 208 ± 13 nm or 106 ± 9 nm, depending on the directions defined by the two different primitive translation vectors of the lattice, as was revealed by AFM (Figure 1a, Figure S2 in Supporting Information File 1). On the one hand, the SEM studies and electrochemical investigations of Au and NIL/Au electrodes resulted in similar data (i.e., NIL modification significantly increased the roughness of the Au surface). On the other hand, contrary to AFM, much higher *f* values were obtained, viz. 1.7 ± 0.1 and 5.5 ± 0.5, for Au and NIL/Au electrodes, respectively, as calculated from electrochemical data. The underestimated *f* values revealed in the AFM studies might be attributed to different factors. For instance, AFM is not sensitive to roughness smaller than that of the tip. Moreover, due to its finite size, it may be that the tip does not reach the bottom of the nanocavities. In this case the corresponding area will not be included in the estimation of the *f* value. Therefore, in our calculations, *f* values from the electrochemical studies were used.

**Figure 2:**
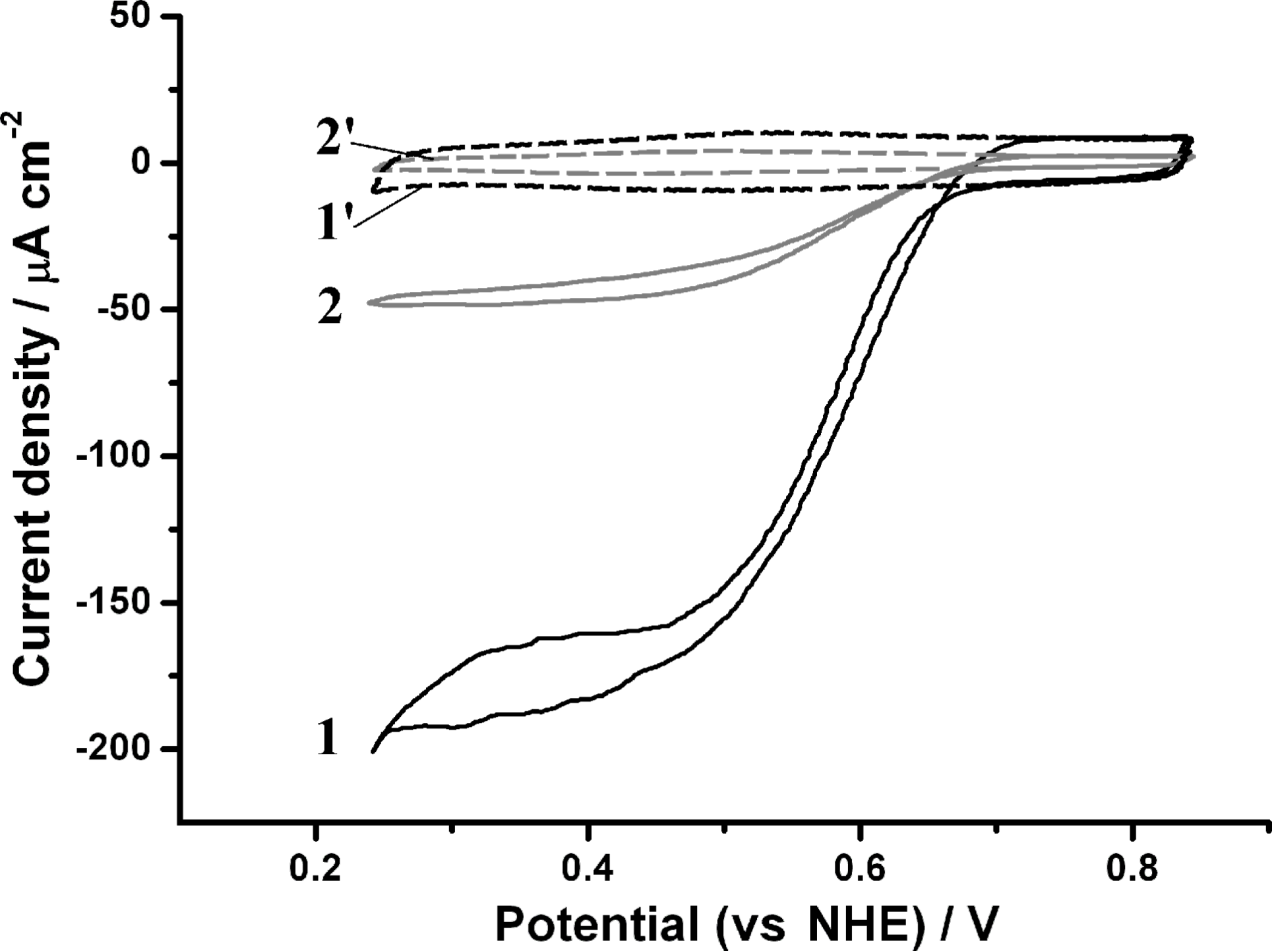
CVs of an NIL/Au electrode (1, 1’) and a Au electrode (2, 2’) modified with *Mv*BOx (1, 2) and without enzyme biomodification (1’, 2’). Conditions: O_2_-saturated PBS, pH 7.4; 20 mV s^−1^ scan rate; second cycle.

Secondly, electrochemical investigations of Au and NIL/Au electrodes were also performed in the O_2_-containing buffer, PBS, over the potential range of 0.0–0.6 V vs SCE (0.24–0.84 V vs NHE). Electrocatalytic reduction of O_2_ was not observed, neither on the Au nor the NIL/Au electrodes (Figure 2, curves 1’ and 2’).

Thus, the biological catalyst, BOx, was immobilised on the Au and NIL/Au electrodes. When the electrochemical measurements of the biomodified electrodes (both BOx/Au and BOx/NIL/Au) were performed in O_2_-saturated PBS, an open-circuit potential (OCP) of O_2_ bio-electroreduction for both types of electrodes was recorded as 0.75 ± 0.03 V vs NHE, that is, very close to the redox equilibrium potential of the O_2_/H_2_O couple under these conditions (0.79 V vs NHE at pH 7.4, 25 °C). This indicates the high bio-electrocatalytic activity of the biomodified electrodes towards O_2_. Indeed, well-pronounced bio-electrocatalytic currents were measured when the CVs were recorded in the potential range of 0.24–0.84 V vs NHE (Figure 2, curves 1 and 2). Maximum bio-electrocatalytic current densities (*j*_max_) of 48 µA·cm^−2^ and 186 µA·cm^−2^ for BOx/Au and BOx/NIL/Au electrodes, respectively, were obtained. When the O_2_ concentration was decreased from 1.2 mM down to 0.25 mM (by saturating the PBS with air instead of oxygen) the maximum current densities of bio-electrocatalytic O_2_ reduction also decreased by factors of 2.7 and 3.2 for the BOx/Au and BOx/NIL/Au electrodes, respectively (Supporting Information File 1, Figures S3 and S4). This result implies the absence of a serious O_2_ diffusion limitation in our electrochemical systems, since in such a case the observed current decrease should be much greater (additional details can be found in Supporting Information File 1). The basic bio-electrocatalytic characteristics of BOx/Au and BOx/NIL/Au electrodes are summarised in Supporting Information File 1, Table S1. Au and NIL/Au electrodes with different surface concentrations of the enzyme (Г) were investigated electrochemically. The maximum bio-electrocatalytic signal was obtained when using 40 μg·mL^−1^ of BOx solution for bio-modification (Supporting Information File 1, Figures S6 and S7). Further increase in enzyme concentration suppressed the current, suggesting the formation of enzyme multilayers. These results are in good agreement with our recent report concerning the interfacial behaviour and activity of BOx immobilised on bare polycrystalline gold surfaces [20].

Thirdly, standard heterogeneous electron transfer rate constants (*k*_0_) and apparent bio-electrocatalytic constants (*k*_cat_^app^, i.e., *k*_cat_ for BOx adsorbed on a Au surface), were calculated based on mathematical modelling (modelled vs experimental curves are presented in Supporting Information File 1, Figure S8). *k*_0_ values for both BOx/Au and BOx/NIL/Au electrodes were found to be equal to 27 s^−1^. It appears that the heterogeneous electron transfer rate for the adsorbed BOx does not depend on electrode nanostructuring. These results are in excellent agreement with our previously published data concerning the influence of gold nanoparticles on enzymatic bio-electrocatalysis [24]. *k*_cat_^app^ values for BOx/Au and BOx/NIL/Au electrodes were found to be 30 and 39 s^−1^, respectively (Supporting Information File 1, Table S1), whereas *k*_cat_ in homogeneous catalysis was measured to be 57 s^−1^ (see above). *k*_cat_^app^ values for BOx/Au and BOx/NIL/Au electrodes were recorded at 30 and 39 s^−1^, respectively (Supporting Information File 1, Table S1), whereas *k*_cat_ in homogeneous solution was 57 s^−1^ (see above). Thus, the *k*_cat_ and *k*_cat_^app^ values do not differ much, suggesting retention of enzymatic activity after physisorption, which is in good agreement with our recently published results [20]. Moreover, it seems as though the biocatalytic activity of adsorbed BOx does not depend on electrode nanostructuring. The overpotential for both BOx/Au and BOx/NIL/Au electrodes was found to be only approximately 0.04 V (see above) and could not be increased further due to thermodynamical constrains. Thus, one cannot explain the significant increase in bio-electrocatalytic signals after NIL just by using *k*_0_ and/or *k*_cat_^app^ values calculated for two different surfaces. However, when the *A*_real_/*A*_geom_ values of Au and NIL/Au electrodes (1.7 vs 5.5, respectively) are compared with the *j*_max_ values measured for BOx/Au and BOx/NIL/Au electrodes (18 µA·cm^−2^ vs 58 µA·cm^−2^ in air-saturated and 48 µA·cm^−2^ vs 186 µA·cm^−2^ in oxygen-saturated PBS), an obvious correlation can be seen. Thus, the experimental results confirm that the improved bio-electrocatalytic currents of BOx/NIL/Au compared to BOx/Au can be simply attributed to an increase in the *A*_real_ of the electrodes.

**Figure 3:**
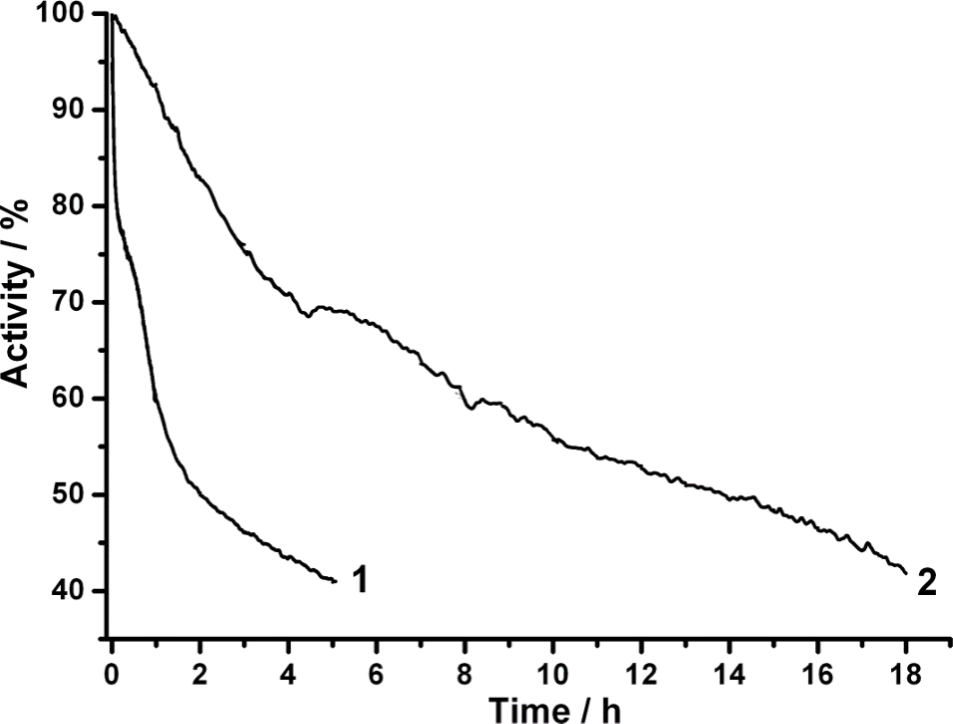
Chronoamperometric responses from a BOx/Au electrode (1) and a BOx/NIL/Au electrode (2). Conditions: O_2_-saturated PBS, pH 7.4; +400 mV potential applied.

Finally, the operational stability of the bio-modified electrodes was also investigated. The half-deactivation times of BOx/Au and BOx/NIL/Au biocathodes were found to be approximately 2 and 14 h, respectively. Thus, the current output of both Au- and NIL/Au-based biocathodes clearly decreased with time (Figure 3).

Since BOx was immobilised on the electrode simply by physical adsorption, this could be attributed to enzyme desorption from the Au surfaces. For this reason, additional qualitative (monitoring of enzyme activity in electrolytes) and quantitative (monitoring of the enzyme layer on Au surface using ellipsometry) studies were performed. In both measurements no indication of BOx desorption from the Au surface was observed. Specifically, no colour difference between the electrolyte (20 mL of PBS, pH 7.4) used in the electrochemical measurements with added enzyme substrates (5 mM ABTS and 0.25 mM O_2_) was observed compared to the control solution (air-saturated PBS containing 5 mM ABTS, which was not in a contact with BOx modified electrodes). However, a well-pronounced colour change developed in electrolytes that were in contact with BOx-modified electrodes during the assays. Moreover, the ellipsometry data clearly showed that after rinsing no desorption of the enzyme took place: the adsorbed amount being approximately constant with a value of approximately 2.6 pmol·cm_real_^−2^ (Supporting Information File 1, Figure S8a). This is close to a dense monolayer of BOx on the Au surface, taking into account that the footprint of the enzyme is about 3 × 10^−13^ cm^2^. Furthermore, it was observed that the thickness of the adsorbed BOx layer gradually decreased, whereas its refractive index (i.e., its density) gradually increased (Supporting Information File 1, Figure S8b,c). This indicates a gradual flattening and compression of the enzyme layer on Au electrodes, which can be considered the origin of the eventual deactivation of the physically adsorbed BOx on bare Au surfaces, as already pointed out in our previous studies [20]. However, based on previous theoretical studies [25], since the *k*_cat_^app^ value for BOx/NIL/Au electrodes is higher compared to BOx/Au (39 and 30 s^−1^, respectively) and no enzyme desorption was observed in our investigations, the significantly improved operational stability of the nanostructured biocathodes compared to “planar” biodevices might be attributed to stabilisation of the enzyme inside nanocavities that are formed by surface nanostructuring using NIL (cf. Figure 1 left (a and c) and right (b and d) images).

## Conclusion

To the best of our knowledge, here we detail one of the first studies in the field of enzyme-based bioelectronics describing high-performance, nanostructured bioelectrodes that can be easily and reproducibly fabricated with industry-scale throughput. Biocompatible, polymer-based, flexible electrodes were fabricated with nanoimprinting, metallisation and biomodification. The very pronounced bio-electrocatalytic reduction of O_2_ in chloride-containing neutral buffers was obtained when the electrodes were biomodified with BOx by simple physical adsorption, which has an irreversible character. Detailed studies of the biocathodes showed that the nanostructured polymer surface provides an approximately 4-fold increase in bio-electrocatalytic current density and an approximately 7-fold increase in the operational stability of the biocathodes compared to planar bioelectrodes. Whereas the bio-electrocatalytic signals are based on an increase in surface area, the improved stability might be attributed to the specific nanogeometry of the fabricated electrodes.

## Experimental

### Chemicals

Unless otherwise specified, all chemicals were purchased from Sigma-Aldrich GmbH (Schnelldorf, Germany). Acetone was purchased from Merck KGaA (Darmstadt, Germany). All solutions were prepared using water purified with the PURELAB UHQ II system from ELGA Labwater (High Wycombe, UK). Nitrogen (N_2_) was additionally purified using gas clean filters from Varian BV (Middelburg, The Netherlands), and O_2_ was obtained from AGA Gas AB (Sundbyberg, Sweden).

### Redox enzyme

*Myrothecium verrucaria* BOx was obtained as a kind gift from Amano Enzyme, Inc. (Nagoya, Japan). The specific activity of BOx, measured to be 140 U·mg^−1^, was determined using 5 mM 2,2'-azino-bis(3-ethylbenzthiazoline-6-sulfonic acid) (ABTS) as an electron donor dissolved in phosphate buffered saline (PBS; 50 mM phosphate buffer containing 0.15 M NaCl), pH 7.4, by measuring O_2_ consumption with a Oxygraph Clark-type electrode from Hansatech, Ltd. (Norfolk, England). Taking into account the molecular weight of the enzyme (close to 59 kDa [20]), the measured specific activity of 140 U·mg^−1^ corresponds to the observed biocatalytic constant (*k*_cat_) of *Myrothecium verrucaria* BOx of about 58 s^−1^. Since ABTS has a very high molar extinction coefficient (ε_418_ = 36000 M^−1^·cm^−1^) the compound was also used for qualitative determination of possible BOx activity in electrolytes [26] due to enzyme desorption from Au surfaces.

### Electrode fabrication and characterisation

#### Fabrication of imprinted substrates

The nanostructured electrodes were fabricated by a thermal NIL process using a nickel stamp purchased from NIL Technologies ApS (Kongens Lyngby, Denmark). The NIL stamp, produced by nickel electroplating, had an array of 100 nm features defined by UV-interference lithography. The stamp had undergone an anti-sticking treatment, resulting in a thin monolayer, self-assembling film of fluorinated alkyl phosphoric acid derivatives, as described in [27]. The pattern transfer step included imprinting using a 6" imprinter machine from Obducat Technologies AB (Lund, Sweden) onto a polymer sheet at 160 °C using an imprint pressure of 50 bar for 120 s and demoulding of the stamp at 115 °C for 40 s. For the imprint material, 20 × 20 cm sheets of the intermediate polymer stamp (IPS^®^) foil, provided by Obducat Technologies AB, was used. The IPS^®^ material is a thermoplastic polymer suitable for thermal imprint with nickel stamps, but its exact composition has not been revealed by the manufacturer. In the current work, the polymer sheet was used to make the nanostructured electrodes with the active Au surface, thus the composition of the nanostructured polymer film had no significance in the current study. Imprinted and non-imprinted sheets of plastic were cut into 10 × 10 mm^2^ pieces with scissors, henceforth called nanostructured and planar sheets, respectively. These were cleaned in acetone at room temperature for 2 min, and dried with N_2_ gas.

#### Thermal evaporation of metal films

All samples were covered with 5 nm of Ti followed by 100 nm of Au by thermal evaporation in a custom built system at low pressure. Titanium wire (99.99+%) was used as the Ti source and was purchased from Goodfellow Cambridge, Ltd. (Huntingdon, England), while the Au nuggets (99.9999%) used as the Au source were purchased from Dahlgren Ädelmetall AB (Malmö, Sweden). The metal films were evaporated at a base pressure of <10^−6^ mbar with an average deposition rate of 1 Å·s^−1^ and 10 Å·s^−1^ for Ti and Au, respectively. The 5 nm thick layer of Ti was deposited to promote the adhesion of the Au layer on the substrates. Supporting Information File 1, Figure S1 shows the fabrication process flow of the nanostructured electrodes.

#### Characterisation of gold electrodes

Surface morphology was studied using scanning electron microscopy (SEM) and atomic force microscopy (AFM). SEM images were taken using a Nova NanoLab 600 Dual Beam focused ion beam and scanning electron microscope (FIB-SEM) from FEI Company (Hillsboro, Oregon, USA). The images were taken with an immersion lens at an acceleration voltage of 30 kV and a beam current of 2.4 nA. AFM images were obtained using a Multimode VIII SPM with a Nanoscope V control unit from Bruker AXS (Santa Barbara, CA, USA). The AFM was operated in the ScanAsyst mode. All images were obtained in air and at room temperature. Triangular silicon nitride cantilevers with a nominal spring constant of 0.4 N·m^−1^ (ScanAsyst Air probes, Bruker AXS) were employed in all measurements. The analysis and processing of the AFM images was performed with the WSxM software package [28]. Image processing consisted of plane subtraction, equalisation, and 3D representation.

The electrochemical measurements of the Au electrodes were performed in 0.5 M H_2_SO_4_ to clean the Au electrodes and to assure uniform Au surfaces on an atomic level, as well as to determine the real (also called microscopic or electrochemically active) electrode areas (*A*_real_) [29]. For this purpose, fabricated Au electrodes were connected as working electrodes to a µAutolab Type III/FRA2 potentiostat/galvanostat from MetrohmAutolab B.V. (Utrecht, The Netherlands) using Au-plated alligator clips (model 3289-2, Pomona Electronics, Everett, WA, USA). These were placed into a standard electrochemical cell with a final electrolyte volume of 20 mL and subjected to an oxidation–reduction cycle in 0.5 M H_2_SO_4_ between 0 and +1.9 V vs NHE at a scan rate of 0.1 V·s^−1^ for 10 cycles. In a three-electrode configuration, a Hg/Hg_2_Cl_2_/KCl_sat_ electrode (SCE, 242 mV vs NHE) and a platinum wire mesh were applied as reference and counter electrodes, respectively. *A*_real_ of the Au electrodes was calculated from the experimentally measured charge (*q*_real_) associated with the Au oxide reduction process performed by running cyclic voltammetry (Supporting Information File 1, Figure S1). A current peak related to the reduction of the Au was integrated to calculate *q*_real_. The theoretical charge density (σ_t_) associated with this process was taken to be 390 µC·cm^−2^ [29].

[1]
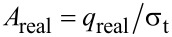


The microscopic roughness factor (*f*) was calculated from Equation 2:

[2]
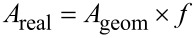


The geometric area (*A*_geom_, also called the two-dimensional projected area) of the electrodes was determined by direct precise geometric measurements using a Vernier caliper from Mitutoyo Scandinavia AB (Upplands Väsby, Sweden).

### Biomodification and investigation of bioelectrodes

#### Modification of gold surface with redox enzyme

Clean Au electrodes with known *f* values derived from electrochemical investigations (see above) were biomodified by simple adsorption of BOx on the electrode surface for 20 min using different solutions of the enzyme: from diluted to very concentrated (from 0.4 µg·mL^−1^ up to 400 µg·mL^−1^ in 10 mM phosphate buffer, pH 7.4) at room temperature (25 °C). It should be emphasised that the electrodes were not allowed to dry out at any time during the modification and electrochemical investigations to avoid possible deactivation due to enzyme dehydration.

#### Studies of biomodified electrodes

The electrochemical measurements were performed in PBS saturated with air and O_2_. Cyclic voltammograms (CVs) and chronoamperograms were recorded using the equipment as described above.

After the electrochemical studies, additional tests concerning residual BOx activity in the electrolytes due to possible enzyme desorption from the electrode surface were performed. For this purpose 20 mL of an electrolyte, which was in contact with BOx-modified electrodes (i.e., after electrochemical measurements), was taken out and replaced with fresh PBS containing 5 mM ABTS (control studies). Simultaneously, 5 mg of ABTS was added to the electrolyte (20 mL of PBS, pH 7.4) used in the electrochemical measurements and possible colour change of the solution was monitored for 1 h. As additional control measurements, air-saturated PBS containing 5 mM ABTS (which was not in a contact with the BOx-modified electrodes) was always prepared and also monitored to estimate the auto-oxidation rate of ABTS by O_2_ under these conditions.

#### Ellipsometry measurements

The adsorption of BOx (40 μg·mL^−1^ solution was used for bio-modification) onto planar Au electrodes (with a roughness factor of about 1.7) was studied in situ by means of null ellipsometry in a similar manner as described in [20]. A thin film, automated ellipsometer (type 43 603-200E, Rudolph Research, Fairfield, NJ, USA) equipped with a xenon arc lamp with a fixed angle of incidence (67.75°) was exploited. In our calculations a refractive index increment of 0.18 mL·g^−1^ with respect to the change in protein concentration (d*n*/d*c*) was used [30].

## Supporting Information

File 1 FileFormat: PDF.
Supplementary data.
The Supporting Information provides additional electrochemical, AFM, and ellipsometry studies of the bioelectrodes, as well as the theoretical basis for the bio-electrochemical investigations and modelling.
