# Supplementary material for: Scalable, high performance, enzymatic cathodes based on nanoimprint lithography
Source: Beilstein J Nanotechnol. 2015 Jun 22;6:1377–84. doi: 10.3762/bjnano.6.142 (PMC4505184; doi:10.3762/bjnano.6.142)
Supplement: File 1 — FileFormat: PDF. Supplementary data. The Supporting Information provides additional electrochemical, AFM, and ellipsometry studies of the bioelectrodes, as well as the theoretical basis for the bio-electrochemical investigations and modelling. [file Beilstein_J_Nanotechnol-06-1377-s001.pdf]

Supporting Information

for

Scalable, high performance, enzymatic cathodes based on nanoimprint lithography

Dmitry Pankratov[1,2], Richard Sundberg[3], Javier Sotres[1], Dmitry B. Suyatin[3,4], Ivan Maximov[3], Sergey Shleev[1,2,5*], and Lars Montelius[3,4]

Address: [1]Biomedical Sciences, Health and Society, Malmö University, 20560 Malmö, Sweden, [2]A.N. Bach Institute of Biochemistry, 119071 Moscow, Russia, [3]Division of Solid State Physics and The Nanometer Structure Consortium (nmC@LU), Lund University, 22100 Lund, Sweden, [4]Neuronano Research Center, Medical Faculty, Lund University, 221 00 Lund, Sweden and [5]Kurchatov NBICS Centre, National Research Centre "Kurchatov Institute", 123182 Moscow, Russia

Email: Sergey Shleev - sergey.shleev@mah.se

* Corresponding author

Supplementary data

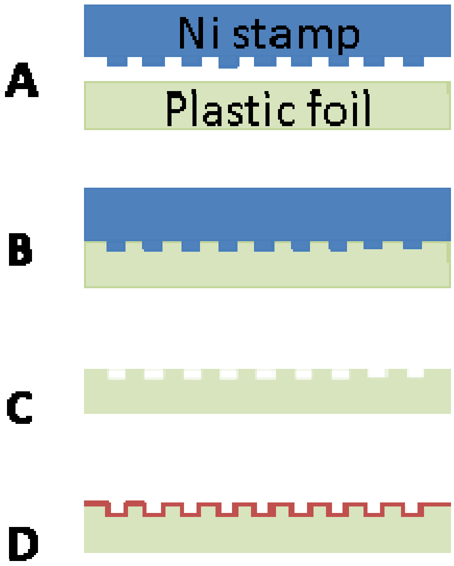

Figure S1: Schematic illustration of the fabrication process of the nanoimprinted plastic electrodes. A) Ni stamp with ca. 100 nm features is used to transfer the pattern into the thermoplastic foil, B) nanoimprint patterning is performed by direct mechanical contact of the stamp and the foil at T equal to 160 °C and pressure of 50 bar, C) after separation of the stamp and the polymer, the stamp nano-pattern is replicated into the polymer, D) 100 nm thick Au layer is thermally evaporated on the imprinted polymer.

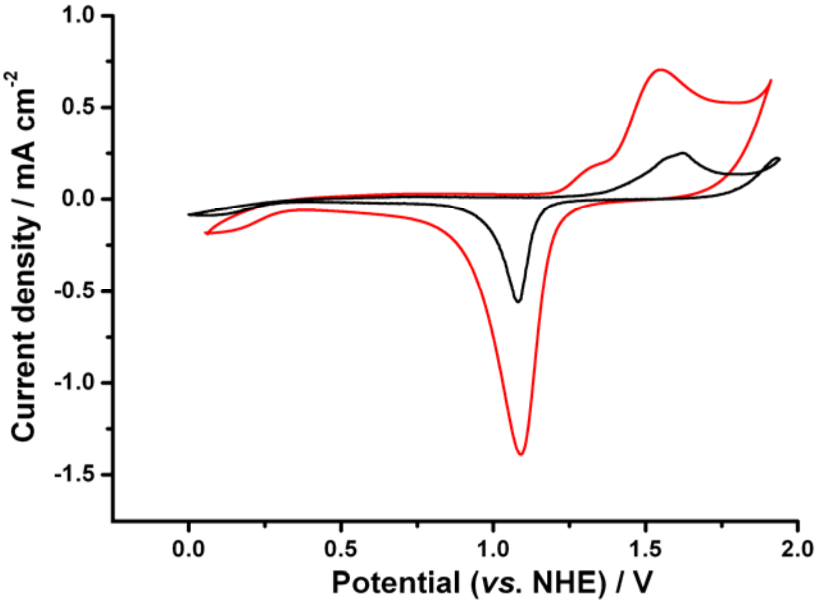

Figure S2: Typical CVs of Au (black curve) and NIL/Au (red curve) electrodes. Conditions: 0.5 M H_2SO_4; 100 mV·s^{-1} scan rate; second cycle.

Additional AFM studies

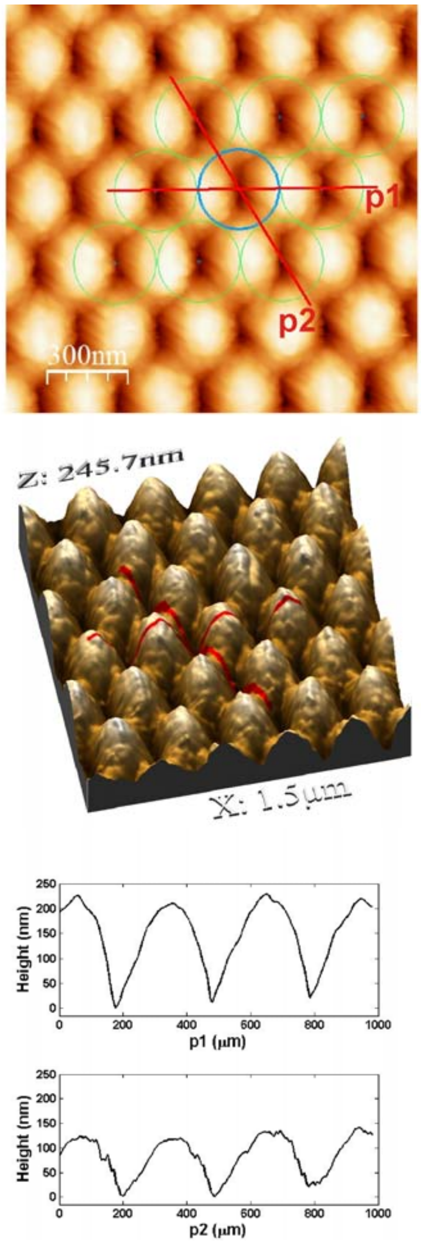

Figure S3: Atomic force microscopy images and corresponding height profiles of nanostructured Au electrodes. The images show 2D and 3D representations of a representative area of the samples. Scan area: 1500 nm × 1500 nm. Colour height scale: 0 nm – 245.7 nm. The height profiles (positions highlighted in red in the topographical images) show the depth of the nano-cavities along the two different directions defined by the primitive vectors of the hexagonal lattice defined by the nanocavities.

Additional bioelectrochemical studies

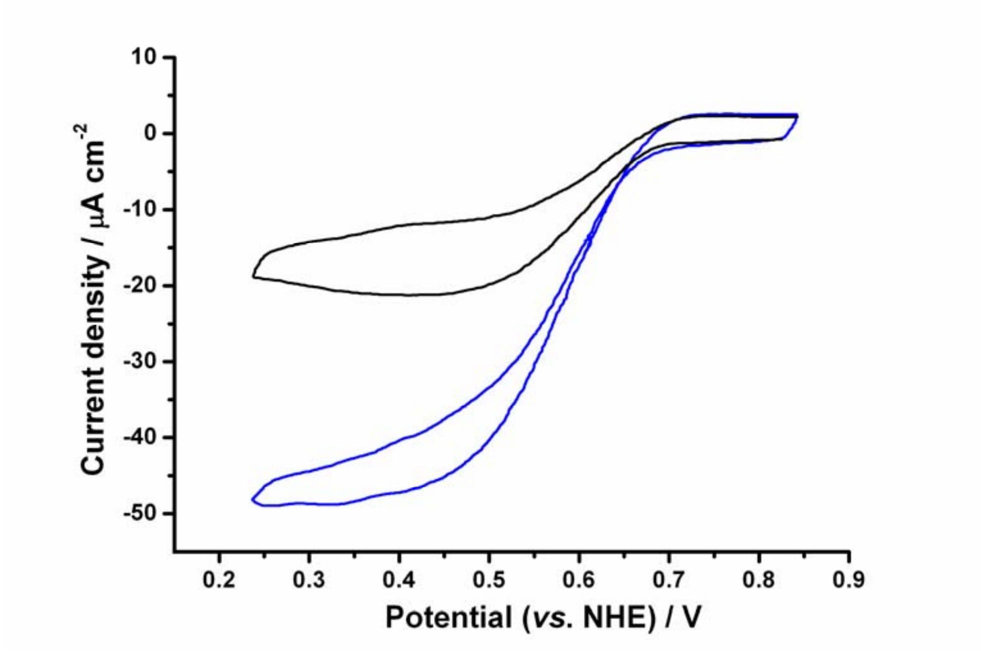

Figure S4: CVs of BOx-modified Au electrodes in air-saturated (black curve) and O_2-saturated (blue curve) buffers. Conditions: PBS; scan rate: 20 mV·s^{-1}; second cycle.

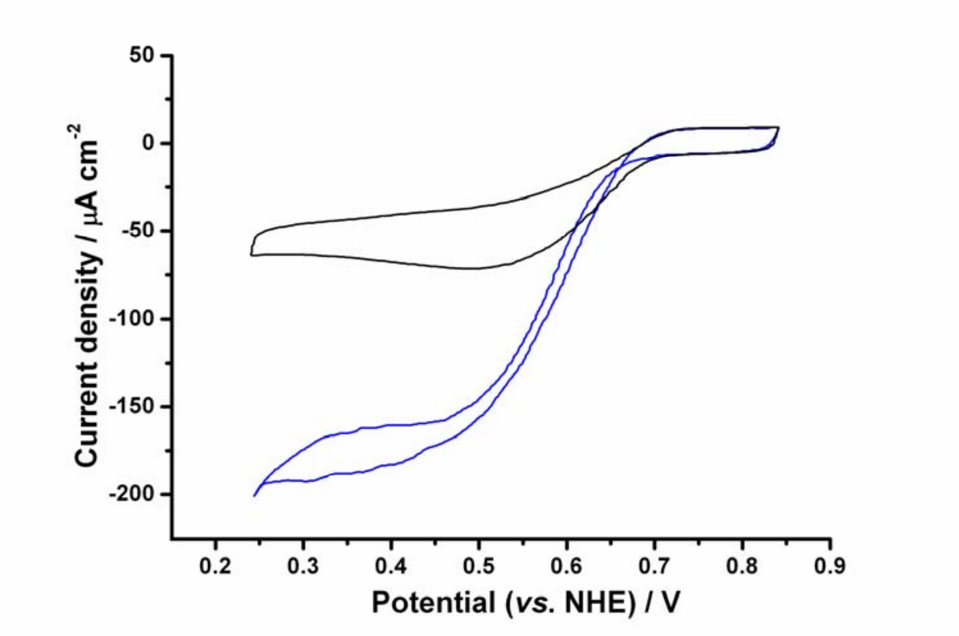

Figure S5: CVs of BOx-modified NIL/Au electrodes in air-saturated (black curve) and O_2-saturated (blue curve) buffer. Conditions: PBS; scan rate: 20 mV·s^{-1}; second cycle.

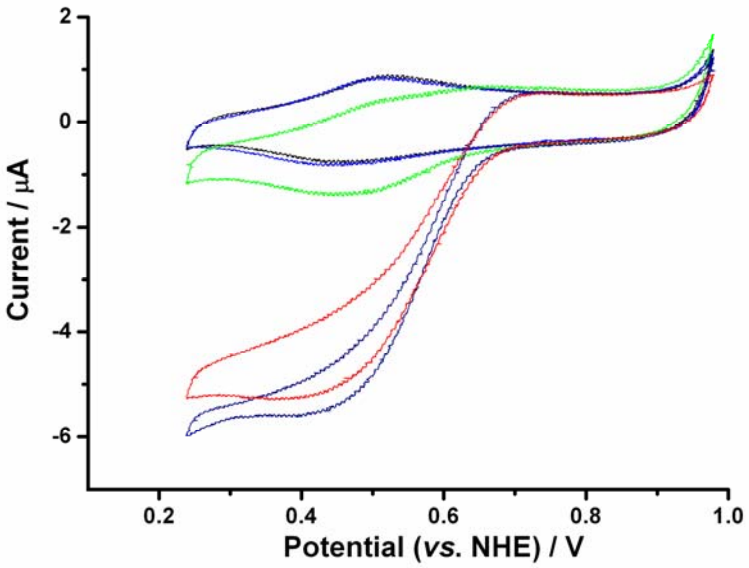

Figure S6: CVs of Au electrode (black curve) and Au electrodes modified with enzyme solutions of different concentrations, μg·mL^{-1}:
0.4 (blue curve), 4 (green curve), 40 (navy curve), and 400 (red curve).
Conditions: O_2-saturated PBS; 20 mV·s^{-1} scan rate; second cycle.

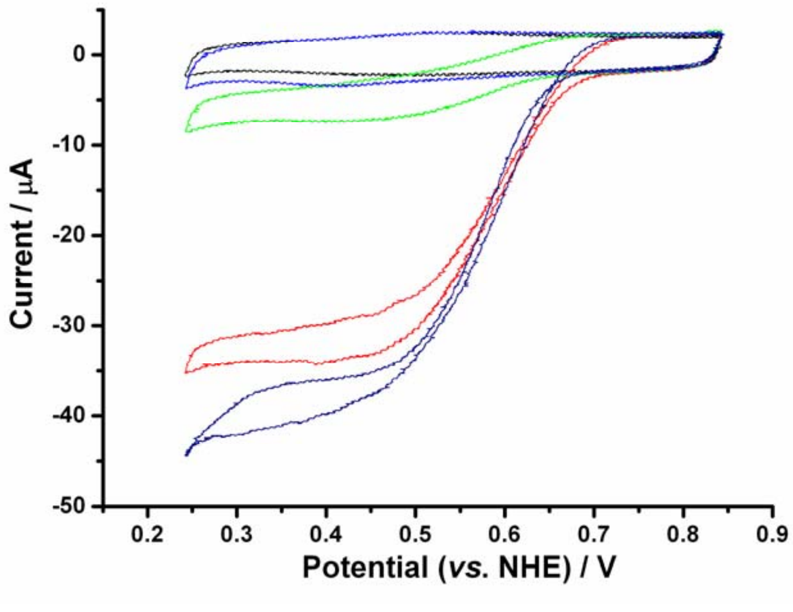

Figure S7: CVs of a non-biomodified NIL/Au electrode (black curve) and NIL/Au electrodes modified with enzyme solutions of different concentrations, μg·mL^{-1}:
0.4 (blue curve), 4 (green curve), 40 (navy curve), and 400 (red curve).
Conditions: O_2-saturated PBS; 20 mV·s^{-1} scan rate; second cycle.

Ellipsometry studies

The time evolution of the adsorbed amount, thickness, and refractive index of BOx layer formed on a planar Au surface were monitored by means of null-ellipsometry (Figure S8). For this, the BOx layer was initially formed by immersing the Au electrodes in a BOx buffer solution at a concentration of 40 μg·mL^{-1} for 60 min (adsorption was also monitored by means of ellipsometry, corresponding to the first 60 minutes of the plots in Figure S8). Then, the ambient solution was replaced with protein-free buffer and the layer monitored for 5 more than hours. Ellipsometry data clearly showed that the adsorbed amount of BOx on the Au surface was approximately constant with a value of ca. 2.6 mg·m_{geom}^{-2} (4.4 pmol·cm_{geom}^{-2} or 2.6 pmol·cm_{real}^{-2}) during the whole process.

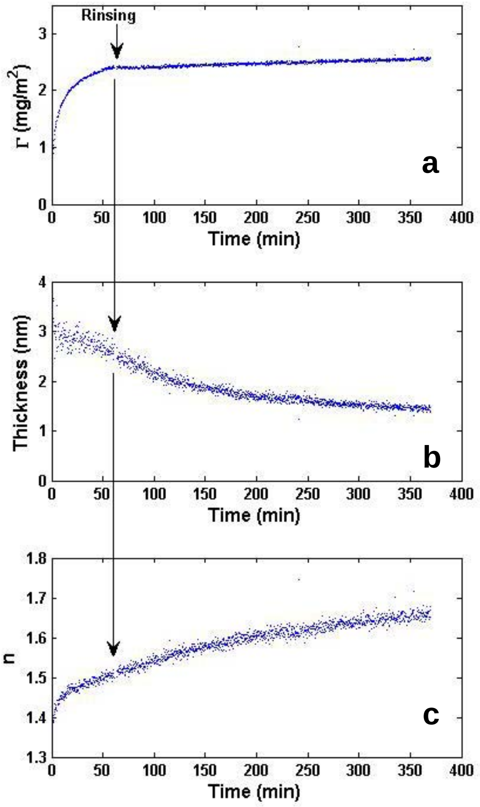

Figure S8: a) Adsorbed amount, **b)** Thickness and **c)** Refractive index corresponding to the formation (initial 60 min), rinsing (following 5 min) and stabilization (following 5 hours) of a BOx layer on a planar Au surface monitored by null-ellipsometry.

Theoretical basis of bioelectrochemical investigations and modelling

For proper mathematical elaboration of bioelectrochemical data, where a bioelectrocatalytic process has a mixed kinetics regime, possible diffusion limitations should be excluded. In general, the rate of a bioelectrocatalytic process can be described by a mixed kinetics equation (Equation S1 [1]).

$$\frac{1}{i} = \frac{1}{i_{ET}} + \frac{1}{i_{cat}} + \frac{1}{i_{s-s}} \qquad \text{(S1)}$$

where, i is the observed current, i_{ET} is the limiting current of the heterogeneous electron transfer (ET; Equation S2; vide infra), i_{cat} is the limiting current of the biocatalytic process (Equation S3), and i_{s-s} is the limiting diffusion current (Equation S4 at ω close to infinity).

$$i_{ET} = nFA_{real}k_0\Gamma \exp\left(\frac{-\alpha n' F(E - E^{0}')}{RT}\right) \qquad \text{(S2)}$$

n' is the number of electrons in the slow electrochemical step, F is the Faraday constant, k_0 is the standard heterogeneous ET constant, Γ is the surface concentration of the enzyme, α is the charge transfer coefficient, E is the electrode potential, $E^{0'}$ is the equilibrium potential of the electrode process, R is the gas constant, and T is the temperature in K.

If intramolecular ET (IET) is not a limiting step in the enzymatic process of O_2 bioelectroreduction (our previous studies of BOx showed that this was the case at pH 7.4 [2]), the bioelectrocatalytic current (i_{cat}) can be expressed as the electrochemical form of the Michaelis–Menten equation:

$$i_{cat} = \frac{nFA_{real}\Gamma k_{cat}^{app}{}_{cat}C_{oxygen}}{C_{oxygen} + K_M} \quad \text{(S3)}$$

where k_{cat}^{app} is the apparent rate constant for the bioelectrocatalytic process and K_M is the Michaelis constant.

The Levich equation (Equation S4) defining steady-state current limited by the transport of substrate molecules at a rotating electrode can be used to estimate mass-transfer limitations:

$$i_{s-s} = 0.62nFD^{2/3}C_{oxygen}A_{geom}\nu^{-1/6}\omega^{1/2} \quad \text{(S4)}$$

here, i_{s-s} is the steady-state diffusion current, n is the total number of electrons, D is the diffusion coefficient of the substrate (19.7 × 10^{-6} $cm^2 \cdot s^{-1}$ for O_2), C_{oxygen} is the bulk concentration of oxygen, O_2 (2.5 × 10^{-7} $mol \cdot cm^{-3}$ and 1.2 × 10^{-6} $mol \cdot cm^{-3}$ of O_2 (air and O_2 saturated solutions, respectively [3]), ν is the kinematic viscosity of the solution (0.01 $cm^2 \cdot s^{-1}$ at 25 °C, a typical value for aqueous solutions), and ω is the angular frequency (in rad s^{-1}).

For a bioelectrocatalytic process strictly limited by O_2 diffusion, direct dependence between C_{oxygen} and j_{max} should be obtained, i.e., j_{max} should decrease by a factor of ca. 5 when the O_2 concentration was decreased from 1.2 mM to 0.25 mM. For electrocatalytic currents, which are limited by reaction kinetics, j_{max} can be simply expressed as the electrochemical form of the Michaelis–Menten equation (Equation S3). When the O_2 concentration was decreased from 1.2 mM down to 0.25 mM in our studies, i.e., when air-saturated buffer was used instead of oxygen, the maximal current

density (j_{max}), which corresponds to bioelectrocatalytic O_2 reduction, decreased by a factor of ca. 3, which follows Equation S3, suggesting just a minor O_2 diffusion limitation. In the present work, recorded CVs were analysed using the kinetic scheme recently elaborated by Climent et al. [4].

The equations defining the currents are as follows:

$$i_{DET} = FA\Gamma[k_1(1-P_1) - k_2P_1)] \quad \text{(S5)}$$

$$k_1 = k_0 \exp\left[-\alpha \frac{F}{RT}\left(E - E_{T_1}^{0'}\right)\right] \quad \text{(S6)}$$

$$k_2 = k_0 \exp\left[(1-\alpha)\frac{F}{RT}\left(E - E_{T_1}^{0'}\right)\right] \quad \text{(S7)}$$

$$i_{cat} = nFA\Gamma \frac{k_{cat}[O_2]}{K_M + [O_2]} \quad \text{(S8)}$$

where k_1 and k_2 are potential dependent DET rate constants defined by the Butler–Volmer formalism (Equations S2, S6, and S7), whereas P_1 represents the fraction of adsorbed BOx molecules with the reduced T1 copper centre.

The maximal bioelectrocatalytic current of O_2 electroreduction, as well as current dependence on the applied potential, can be described by the following summarised equation:

$$j = \frac{j_{max}}{1 + \exp\left[\frac{F}{RT}\left(E - E_{T1}^{0'}\right)\right] + \frac{k_5}{k_0}\exp\left[\frac{\alpha F}{RT}\left(E - E_{T1}^{0'}\right)\right]} \quad \text{(S11)}$$

Using this equation, modelling studies of obtained bioelectrocatalytic signals were performed (Figure S9) and basic parameters of bioelectrocatalytic reduction of O_2 were calculated (Table S1), assuming K_M and Γ are equal to 0.2 mM and 2.6 pmol·cm_{real}^{-2},

respectively. For the calculations, the K_M value was taken from our previous studies of the enzyme immobilised on bare polycrystalline Au surfaces [5], whereas the Γ value was obtained from the ellipsometry studies (vide supra).

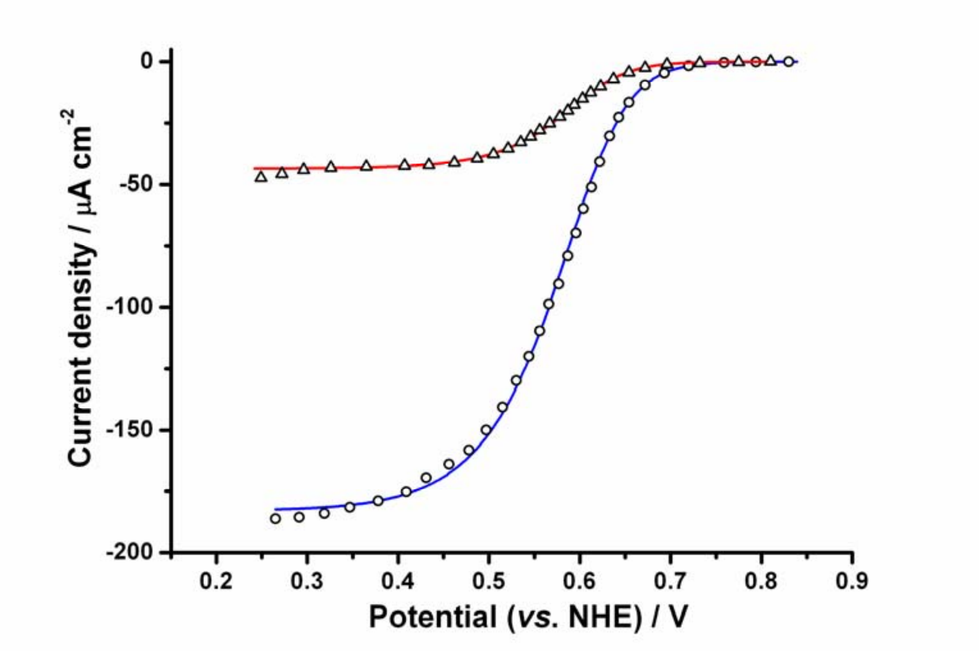

Figure S9: Experimental versus modelled voltammograms of Au (triangles) and NIL/Au (circles) biocathodes.
(points – experimental data, lines – modelled curves).
Conditions: O_2-saturated PBS; 20 mV·s^{-1} scan rate; second cycle;
an enzyme solution with a concentration equal to 40 μg·mL^{-1} was used for the bio-modification.

Table S1: Calculated bio-electrocatalytic parameters of Au and NIL/Au bioelectrodes.

Parameter	Au	NIL/Au
Maximal bio-electrocatalytic current density (j_{cat}; μA·cm^{-2}; air-saturated buffer)	18 ± 3	58 ± 6
Maximal bio-electrocatalytic current density (j_{cat}; μA·cm^{-2}; O_2-saturated buffer)	48 ± 5	186 ± 11
k^{app}_{cat} (s^{-1})	30 ± 4	39 ± 5
k_0 (s^{-1})	27 ± 3	27 ± 3

References

1. Sucheta, A.; Cammack, R.; Weiner, J.; Armstrong, F. A. *Biochemistry* **1993,** *32,* 5455–5465. doi:10.1021/bi00071a023
2. Shleev, S.; Andoralov, V.; Falk, M.; Reimann, C. T.; Ruzgas, T.; Srnec, M.; Ryde, U.; Rulíšek, L. *Electroanalysis* **2012,** *24,* 1524–1540. doi:10.1002/elan.201200188
3. Truesdale, G. A.; Downing, A. L. *Nature* **1954,** *173,* 1236. doi:10.1038/1731236a0
4. Climent, V.; Zhang, J.; Friis, E. P.; Østergaard, L. H.; Ulstrup, J. *J. Phys. Chem. C* **2012,** *116,* 1232–1243. doi:10.1021/jp2086285
5. Pankratov, D.; Sotres, J.; Barrantes, A.; Arnebrant, T.; Shleev, S. *Langmuir* **2014,** *30,* 2943–2951. doi:10.1021/la402432q
